# Supplementary material for: Spatial and temporal localization of SPIRRIG and WAVE/SCAR reveal roles for these proteins in actin-mediated root hair development
Source: Plant Cell. 2021 Apr 20;33(7):2131–48. doi: 10.1093/plcell/koab115 (PMC8364238; doi:10.1093/plcell/koab115)
Supplement: koab115_Supplementary_Data [file koab115_supplementary_data.zip › tpc.00196.2021-s01.pdf]

Supplemental Data. Chin et al. (2021). Spatial and temporal localization of SPIRRIG and WAVE/SCAR reveal roles for these proteins in actin-mediated root hair development. Plant Cell.

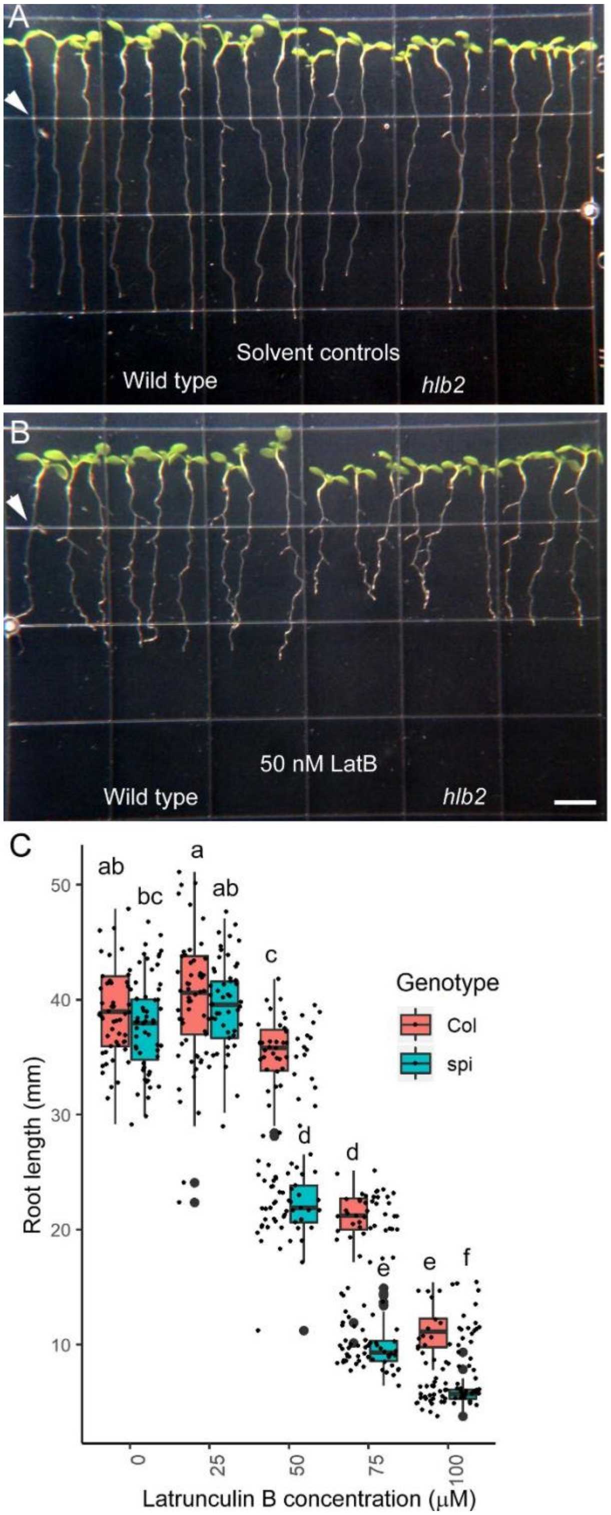

Supplemental Figure 1. Primary Root Growth of *hlb2* Seedlings is Hypersensitive to LatB. (Supports Figure 1)
(A) to **(B)** Representative images of wild type and *hlb2* 6 days after transplanting 3-d-old seedlings on solvent control **(A)** and 50 nM LatB plates **(B)**. Arrowheads indicate the position of the root tip during seedling transplant. Bar = 5 mm.
(C) Dose–response analyses of primary root length of wild type and *hlb2* 6d after transplanting 4-d-old seedlings to a range LatB concentrations. Statistical significance was determined by one-way ANOVA. Means (n = 40-54 roots) ± SE. Different letters are significantly different ($P < 0.05$, Tukey’s test).

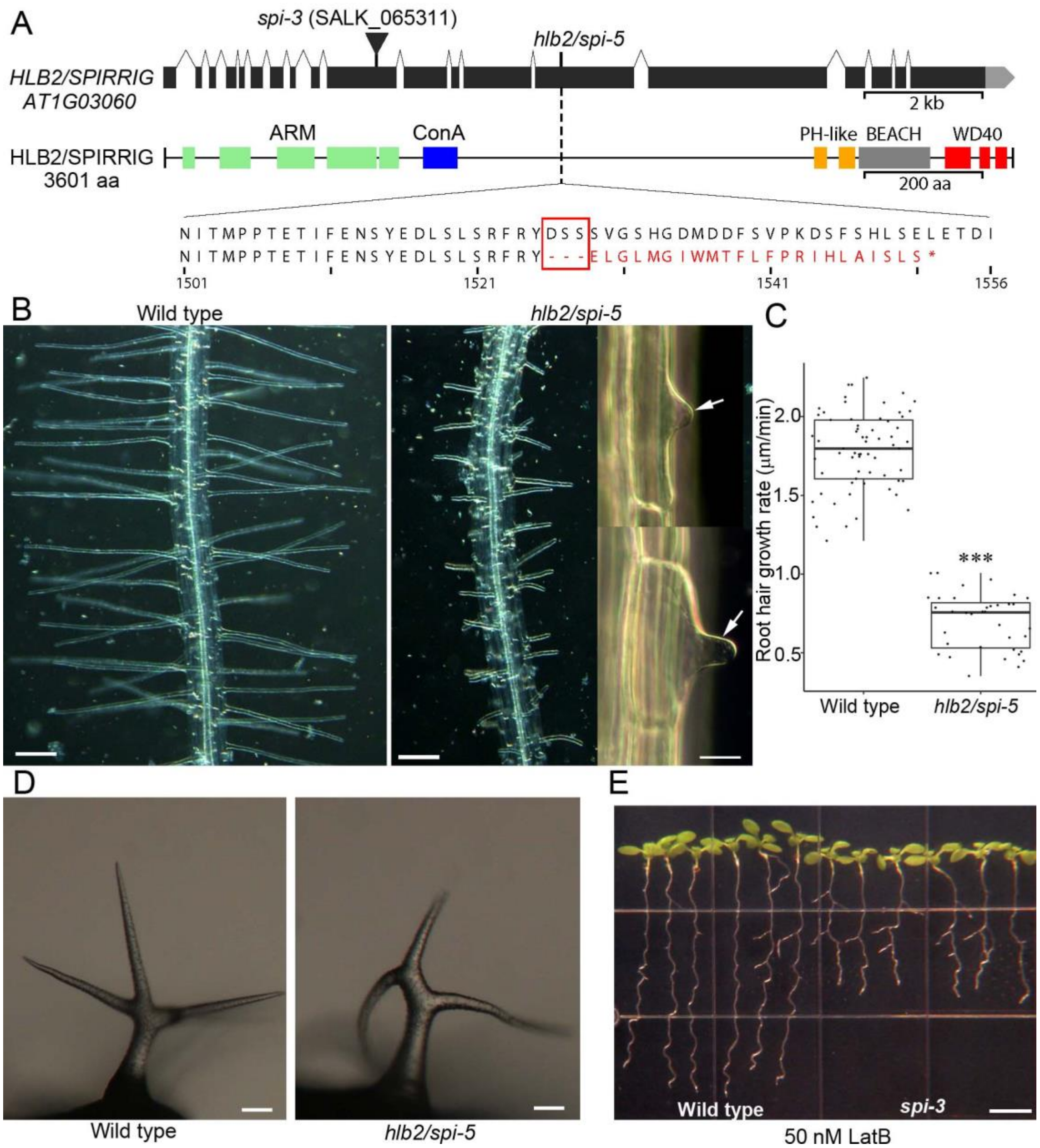

Supplemental Figure 2. *HLB2* Encodes the Beach Domain-Containing Protein, SPIRRIG (SPI). (Supports Figure 1)

(A). Schematic diagram of *HLB2/SPI* gene and the corresponding domain organization of its protein product showing the position and nature of the *hlb2/spi-5* mutation. Inverted triangle marks the position of the mutation in *spi-3*. ARM=Armadillo repeat; ConA=Concanavalin A; PH = Pleckstrin Homology; BEACH= Beige and Chediak Higashi.

(B) Root hairs of *hlb2/spi-5* are significantly shorter than wild type. Some root hairs of *hlb2/spi-5* form small bulges (arrows) and are unable to transition to tip growth. Bars for low magnification image = 50 μm; Bar for high magnification image = 10 μm.

(C) Box plot of root hair growth rate in wild type and *hlb2/spi-5*. Box limits indicate 25th percentile and 75th percentile, horizontal line is the mean and whiskers display min and

max values. Asterisk (***) indicates statistical significance ($p<0.0001$) as determined by Student's T-test.
(D) *hlb2/spi-5* exhibit mild trichome defects manifested primarily as crooked branches. Bar= 50 μm.
(E) Like *hlb2/spi-5*, primary roots of *spi-3* is hypersensitive to LatB. Bar = 5 mm

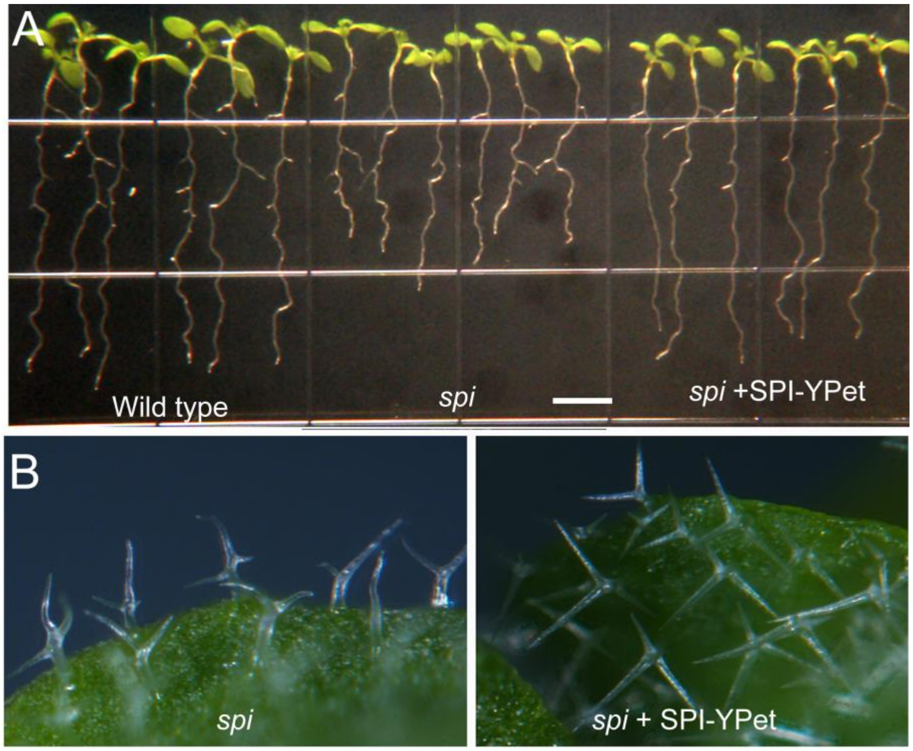

Supplemental Figure 3. *SPIpro:SPI-YPet* complements the primary root hypersensitivity to LatB (**A**) and distorted trichome phenotypes (**B**) of *spi*. (Supports Figure 1)

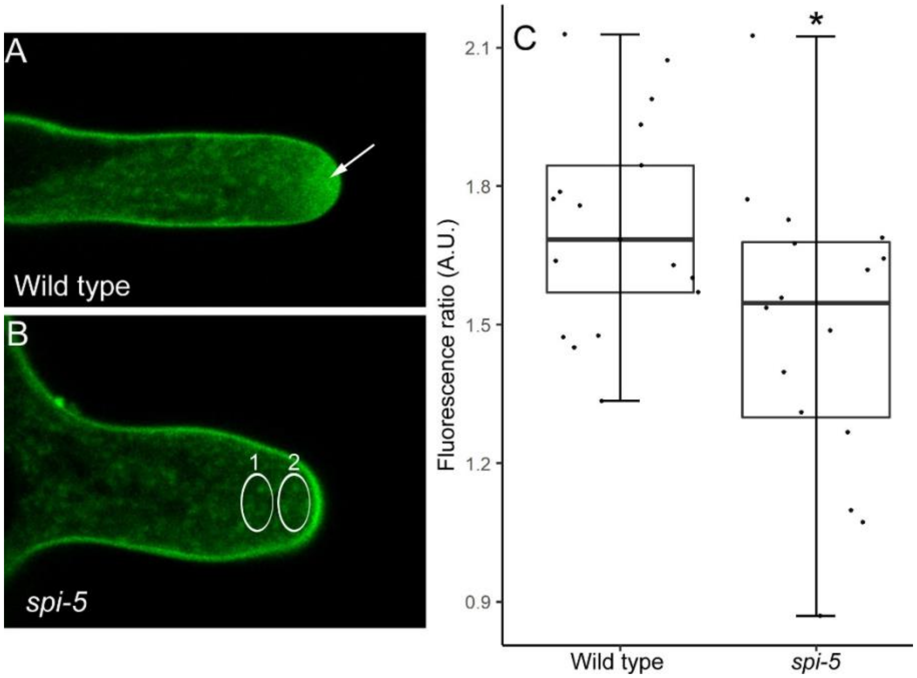

Supplemental Figure 4. FM-1-43 uptake assays in wild-type and *spi* root hairs. (Supports Figure 4)

(A) and **(B)** Distinct FM-1-43 fluorescence accumulates at the tips of wild type (arrows), but not *spi* root hairs

(C) Quantification of FM1-43 gradients in wild type and *spi* root hair tips. Fluorescence ratio was obtained by marking oval regions of interests as shown in panel **B** and dividing 2 over 1. Box limits indicate 25th percentile and 75th percentile, horizontal line is the mean and whiskers display min and max values. Asterisk (*) indicates statistical significance (p=0.02) as determined by Student's T-test. n=16 root hairs from at least 10 independent seedlings.

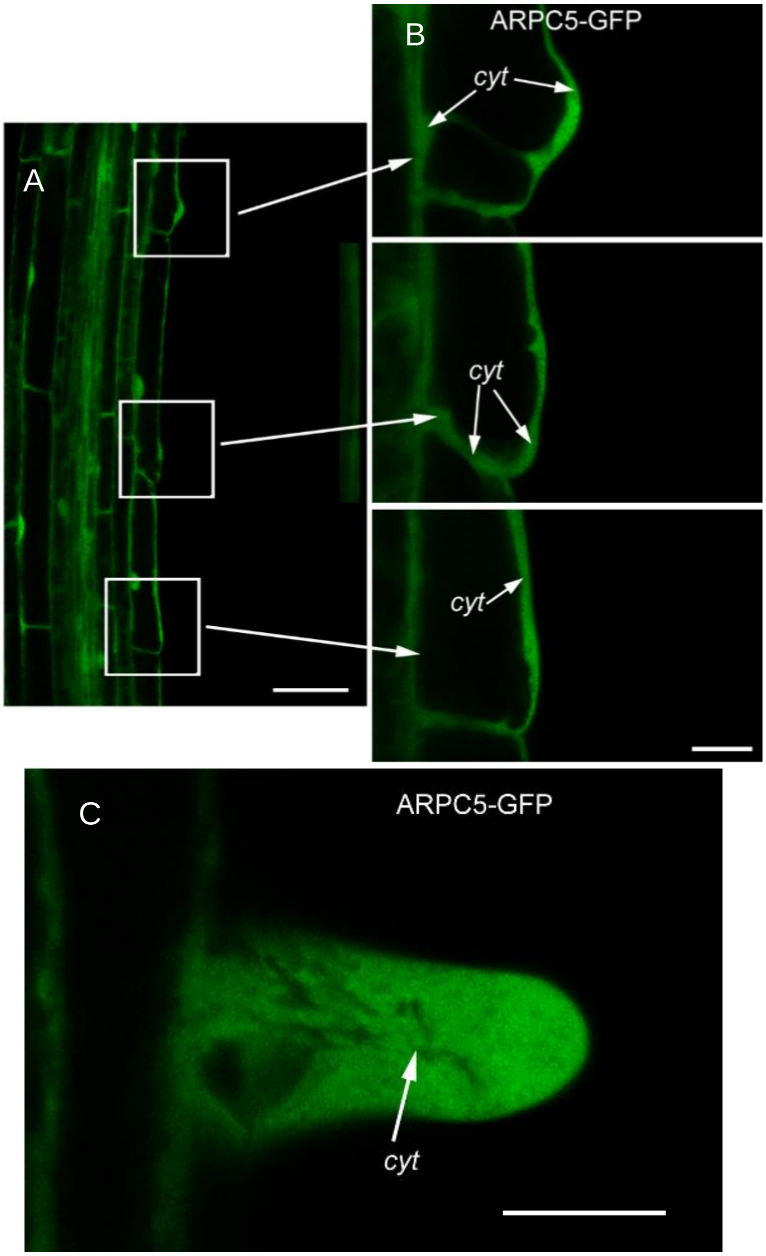

Supplemental Figure 5. ARP2/3 Pathway Does Not Mark the Root Hair Initiation Domain. (Supports Figure 5)
(A) to **(C)** ARPC5-GFP localization in the root maturation zone and elongating root hairs. ARPC5-GFP is located in the cytoplasm (*cyt*) of trichoblasts and did not show distinct gradients in fluorescence signal in the root hair initiation domains, root hair bulges **(A,B)** and elongating root hairs **(C)**. Bars = 10 µm.

Supplemental Table 1. Statistical test results for *t*-test.

	Degrees of freedom	t- value	P-value
Figure 2D Control vs BFA treated	1	5.873	< 0.0001
Figure 2F Wild type vs *spi-5*	1	6.378	< 0.0001
Figure 3P Wild type vs *spi-5*	1	5.215	< 0.0001
Figure 6E *brk1* vs *spi-5*	1	3.054	< 0.001
Figure S2C Wild type vs *spi-5*	1	23.33	< 0.0001
Figure S4C Wild type vs *spi-5*	1	2.368	<0.05

Supplemental Table 2. Statistical test results for ANOVA.

	Sum of squares	Degrees of freedom	Mean Squares	F-value	P-value
Figure 3Q	21.7198	3	7.2399	158.45	<0.0001
Figure S1C	3292	4	823	72.163	<0.0001
