# Supplementary material for: Spatial and temporal localization of SPIRRIG and WAVE/SCAR reveal roles for these proteins in actin-mediated root hair development
Source: Plant Cell. 2021 Apr 20;33(7):2131–48. doi: 10.1093/plcell/koab115 (PMC8364238; doi:10.1093/plcell/koab115)
Supplement: koab115_Supplementary_Data [file koab115_supplementary_data.zip › tpc.00196.2021-s11.docx]

Supplemental Data. Chin et al. (2021). Spatial and temporal localization of SPIRRIG and WAVE/SCAR reveal roles for these proteins in actin-mediated root hair development. Plant Cell.

**Supplemental Movie S1** –Low Magnification Time-Lapse Confocal Microscopy of SPI-YPet of Several Rapidly Elongating Root Hairs, Total Elapsed time is 2 h with images capture every 5 min. (Supports Figure 1)

**Supplemental Movie S2.** Time-Lapse Confocal Microscopy of SPI-YPet in a Rapidly Elongating Root Hair. Total elapsed time of the movie is 50 min with images captured every 5 min. (Supports Figure 1)

**Supplemental Movie S3.** Time-Lapse Confocal Microscopy of SPI-YPet in a Root Hair Bulge Transitioning to Tip Growth. Total elapsed time of the movie is 35 min with images captured every 5 min. (Supports Figure 1)

**Supplemental Movie S4.** Time-Lapse Confocal Microscopy of SPI-YPet in a Root Hair during Termination of Tip Growth. Total elapsed time of the movie is 60 min with images captured every 5 min. (Supports Figure 1)

**Supplemental Movie S5.** Spinning-Disc Confocal Microscopy of SPI-YPet in a Elongating Root Hairs of Wild type and *spi-5*. Total elapsed time of the movie is 120 min with images captured every 1 sec. (Supports Figure 3)

**Supplemental Movie S6.** Spinning-Disc Confocal Microscopy of a Rapidly Elongating Root Hair Co-Expressing SPI-YPet and mRuby3-Lifeact. Total elapsed time of the movie is 5 min with images captured every 3 sec. (Supports Figure 4)

**Supplemental Movie S7.** Time-Lapse Confocal Microscopy of BRK1-YFP Root Hair Bulge Transitioning to Tip Growth. Total elapsed time of the movie is 35 min with images captured every 5 min. (Supports Figure 5)

**Supplemental Movie S8.** Time-Lapse Confocal Microscopy of a Root Hair Bulge Transitioning to Tip Growth and Co-Expressing SPI-YPet and mRuby3-Lifeact. Total elapsed time of the movie is 70 min with images captured every 10 min. (Supports Figure 6)

**Supplemental Movie S9.** Time-Lapse Confocal Microscopy of a *spi* Root Hair Expressing BRK1-YFP. Total elapsed time of the movie is 80 min with images captured every 10 min. (Supports Figure 6)
