# Supplementary material for: Spatial and temporal localization of SPIRRIG and WAVE/SCAR reveal roles for these proteins in actin-mediated root hair development
Source: Plant Cell. 2021 Apr 20;33(7):2131–48. doi: 10.1093/plcell/koab115 (PMC8364238; doi:10.1093/plcell/koab115)
Supplement: koab115_Supplementary_Data [file koab115_supplementary_data.zip › tpc.00196.2021-s13.pdf]

THE PLANT CELL Peer Review Report

Spatial and Temporal Localization of SPIRRIG and WAVE/SCAR Reveal Roles for These Proteins in Actin-Mediated Root Hair Development

Sabrina Chin, Taegun Kwon, Bibi Rafeiza Khan, J. Alan Sparks, Eileen L. Mallery, Daniel B. Szymanski, and Elison B. Blancaflor

Corresponding authors: Elison B. Blancaflor elisonwhiteflower@gmail.com *and Sabrina Chin* schin7@wisc.edu.

Review timeline:

TPC2020-RA-00942	Submission received:<br>1st Decision:	Nov. 13, 2020<br>Dec. 22, 2020 *manuscript declined*
**TPC2021-RA-00196**	Submission received:<br>1st Decision:	March 10, 2021<br>March 15, 2021 *accept with minor revision*
**TPC2021-RA-00196R1**	1st Revision received:<br>2nd Decision:<br>Final acceptance:	March 31, 2021<br>March 31, 2021 *acceptance pending, sent to science editor*<br>April 13, 2021

REPORT: (The report shows the major requests for revision and author responses. Minor comments for revision and miscellaneous correspondence are not included. The original format may not be reflected in this compilation, but the reviewer comments and author responses are not edited, except to correct minor typographical or spelling errors that could be a source of ambiguity.)

TPC2020-RA-00942 1st Editorial decision – *declined* Dec. 20, 2020

As you will note, the reviewers expressed enthusiasm for the novelty of your findings, which was also shared among the editors. Therefore, during the post-review consultation session, we agreed that if you could address the major points raised by the reviewers, we would welcome a resubmission. This may be treated as a new submission, but we would attempt to use at least some of the same reviewers. Nevertheless, reviewers will be asked to assess as a new manuscript (i.e. are the claims fully supported by the data and do the results presented move the field forward?).

There was a consensus amongst reviewers and editors that in the current form, the manuscript poses a model that is too speculative and is not fully supported by the data. Note that reviewer 3 comments that some of the observed effects may be indirect. Moving forward, we suggest that you consider two possibilities: 1) resubmit as a regular article with additional data to strengthen the conclusions, or 2) resubmit as a 'Breakthrough Report' focusing on the exciting new localization results. The genetics, which are more difficult to interpret, could be included as supplemental material or removed. In either case, we would urge you, if you choose to include a model, to ensure that there is substantially more support for the model.

-- Reviewer comments:
Reviewer #1 (Comments for the Author):
This manuscript addresses the open question of how actin and endomembrane systems coordinate for tip growth in root hairs, focusing on the SPI protein and the Arp2/3 actin polymerization pathway. The topic is interesting, and the data are, for the most part, well presented. One major area of concern, however, is the integration of data from various mutants into the model presented in Figure 8. The parts of the model that are well supported are:
1. SPI plays a role in tip growth in root hairs.
2. SPI localizes to the root hair tip.
3. SPI localization is temporally anti-correlated with BRK1 localization, and BRK1 remains localized to the growing tip in the absence of SPI.
4. Root hair positioning depends on both SPI and SCAR/WAVE activity, and BRK1 localizes to the RHID.
5. SPI and the Arp2/3 complex both contribute to root hair extension (although it is less clear whether and how SCAR/WAVE plays a role here).
The other conclusions are based on a *brk1* phenotype that is less convincing. In figure 6A, the *brk1* root hairs look

just as long as WT, if not longer, and it appears there may be more root hairs in the mutant. Is this image representative? In the quantification (6B), how many plants did the root hairs come from? It seems likely that a few outliers are driving the significance for the difference between WT and *brk1* root hairs. It would be helpful to know if these outliers were found in multiple individuals, or were from the same plant. If *brk1* root hairs are not really different from WT root hairs, the authors might consider altering their conclusion that BRK1 is involved in tip growth: Line 417: "Taken together, our results indicate that SPI is epistatic to BRK1 for root hair planar polarity and tip growth." (To be clear, this concern does not apply to the polarity phenotypes quantified in Fig. 6C which appear robust.) An alternative hypothesis could be that the SCAR complex is involved in root hair positioning, but not in determining root hair length. This interpretation is consistent with localization data shown in Figure 5A, where SCAR localization is highest during bulge formation and then drops precipitously during root hair growth.

Finally, the model proposed in Figure 8 includes a lot of details for which the authors have yet to support with data. The authors are up front about the speculative nature of the model, but a simpler model that includes only proteins for which they (or others from the literature) have data seems more appropriate. The authors could also consider showing two models: one showing how SPI might interact in the SCAR pathway, and a second showing their alternative hypothesis that SPI acts in a parallel pathway.
Other major points that should be addressed:

1) Data Quantitation:
There are a number of places in the paper where the conclusions would be strengthened if the authors were to provide a quantitative analysis of their data:

Fig. 1: What percentage of elongating root hairs show robust SPI-YPet localization at the tip? The localization is very clear: the question is do the authors see this phenotype in all plants, or in just one or two? Presumably the authors saw the same phenotypes across multiple root hairs and plants, but the data should be provided.

Fig. 2: Only one example of a root hair treated with BFA is shown. This is a key result and it seems appropriate to convey how variable this phenotype is. Two additional examples from the elongation zone are shown, but these phenotypes seem qualitatively different than the single root hair example (the puncta are much more obvious in the elongation zone). The authors should quantitate the frequency and intensity of the BFA phenotype in root hairs.

Fig 3Q: Co-localization of SPI and F-actin is an important finding, yet the authors show only a single example which is not very compelling: actin intensity is seen throughout the cell, so the only way the image could show lack of co-localization would be a depletion of actin where SPI-YFP is. At minimum, additional examples should be provided, but quantitation of the phenotypes would be preferable.

Fig. 5: The timecourse data do not indicate how frequently the authors see the patterns shown. Presumably the authors saw the same phenotypes across multiple root hairs and plants, but the data should be provided. At minimum, additional examples should be provided, but quantitation of the phenotypes would be preferable.

2) Statistics and data visualization:
The authors should provide additional statistical information, including:

There is something unusual going on with the error bars (standard error) for some of the samples shown Figures 6B and 7C. These graphs are plotted on a log scale, but the error of some of the samples are the same height above and below them mean. On a log scale, we would expect the error to be shorter above the mean and longer below to reflect the same number.

N is stated in many places throughout the paper as the number of root hairs analyzed. Are these from a single plant or across multiple individuals?

Fig. 7 is central to the take home message of the paper, and the authors should include all the data points as a bee swarm plot or similar, particularly for panel B as the difference between WT and *brk1* is central to their model.

Minor points that the authors may want to consider:

Figure 1:
It is difficult to see some of the images in B and C. An inverted LUT, a black and white LUT and/or an outline of the root would be helpful for orientation.
Fig. S1C: please show all the data points
Some of the text and arrows in the figures are very small (e.g. axis labels in 1D).
In figures 1C and D, as well as 3O and 3P, why did the authors choose to normalize the fluorescence intensity to an area outside of the root hair rather than an intracellular region as for Fig. 2F? The latter seems better.

Figure 2:
Fig. 2D: The black type on DIC images is very hard to read. White would be preferable
Fig. 2F: Please show all the data points.

Figure 3: Fig. 3P: how were the root hair tips selected to be analyzed? This seems important as the phenotype changes with the age of the root tips.

Figure 4: Fig. 4: it is Confusing to have three asterisks mean two different things in one figure. Perhaps an arrow head of a second color could be used in fig. 4E?

Figure 6: What about calling Fig. 6 "SPI is epistatic to BRK1 in root hair development" as that is what the data show? It would be useful for the reader to explain the choice of pairwise Kolmogorov-Smirnov Z test. Seems reasonable given the difference in the shapes of the distribution for phenotypes, but useful to explain choice of test.
Fig. S1C: It would be preferable to show all the data points.

Line 521: "Taken together, these results indicate that actin-mediated regulation of polarized cell growth by BEACH domain-containing proteins is likely to be conserved across animals and plants." Because cell growth in plants and animals is so different, this statement seems a bit of a stretch. Perhaps the authors could consider saying "actin-mediated regulation of cell polarity".

Line 396: "SPI is antagonistic to BRK1 during the transition from root hair initiation to rapid tip growth." I think the authors mean SPI is antagonistic to BRK1 localization. If so, this should be clarified as without adding "localization", the inference is that SPI is antagonistic to BRK1 function, and the immediately preceding data does not show this.
Typo on line 105, "inactive closed"

Potential typo on line 110, the subunit is commonly referred to as abi (or abi1)

Reviewer #2 (Comments for the Author):
This manuscript provides important new insights into the mechanisms that mediate tip growth of root hairs in Arabidopsis, and by extension all flowering plants. The authors describe the isolation of a new allele of the *SPIRRIG* (*SPI*) gene based on its heightened sensitivity to the actin disruptor latrunculin B. A functional tagged version of the SPI protein localized to what appear to be post-TGN vesicles at the tips of actively growing root hairs. Tip-focused actin filaments were depleted in the mutant. Interestingly, members of the W/SCR complex (BRK1 and SCAR2) localized to the root hair initiation domain but were lost from the tip of root hair bulges during transition to tip growth

when SPI appears. This apparent antagonistic behavior of these proteins was also supported by the continued presence of BRK1-YFP at the tip of *spi* root hairs. Finally, the authors analyzed the genetic interactions between *spi* and *brk1* as well as several mutants of members of the ARP2/3 complex. These experiments revealed that *spi* was epistatic to *brk1* for root hair phenotypes but showed different interactions with *arp2/3* mutants.

This is a very interesting and well-written manuscript that uncovers several new mechanisms that were previously unknown. The discovery that SPI functions by regulating actin filaments independently of ARP2/3 is particularly valuable. The experimental results for the most part strongly support the authors conclusions. I have only a few comments and suggestions that the authors may want to consider in order to further strengthen their manuscript.

Major Points:
1. (line 273 and 304 ff) The authors mention slow-growing root hairs in *spi* mutants. It would be helpful to characterize these growth rates so they could be compared to, for example, the rates shown in Figure 1D. This analysis could also be extended to an explicit comparison on growth rate and actin fluorescence. The authors already have data on the latter (Figure 3P).
2. (line 390) The observation of prolonged persistence of BRK1-YFP in elongating root hairs of the *spi* mutant is very interesting. I am wondering whether the *spi* root hairs can only grow when BRK1 is maintained at the root hair tip. A prediction from this hypothesis would be that all actively growing *spi* root hairs have BRK1-YFP signals at their tip, and that there may be a positive correlation of this signal with growth rate (unlike in WT). This test could indicate whether ARP2/3 activation could compensate for the loss of SPI. I assume the authors already have the necessary to data to perform such an analysis.
3. (line 275) The authors present data in Figure 2D that suggest that a defect in accumulation of exocytic vesicles at the root hair tip of *spi* mutants. This is difficult to reconcile with the ongoing (albeit slow) growth of these root hairs. It would be helpful to test with another marker such as FM4-64 whether the accumulation of vesicles at the tip of growing *spi* root hairs is really gone.
4. (line 457) I don't think the statements that *arp2, arp3*, and *brk1* "leaned more toward wild-type sensitivity" and that the *spi brk1* double mutant "was more similar to spi" are justified. The single mutants have growth reductions that are about half-way between WT and spi, and the *spi brk1* growth inhibition seems to be additive of the single mutants. Based on the data in Supplemental Figure 6B, it seems that *spi* is epistatic to *arp2* and *arp3* for LatB inhibition of primary root growth, but that *spi* and *brk1* function additively. These are different genetic interactions from those found for the root hair phenotypes, but this does not invalidate the conclusions of the authors for root hair growth.

Minor Points:
1. (line 266) The authors speculate that SPI protein arrives at the root hair tip on post-Golgi vesicles. If this were the case, then I would expect to see some punctate labeling along the shank of the root hair. In most of the images provided these predicted spots are not readily discernible. Could it be that the SPI protein only associates with the vesicles once they reach the tip (and that this "maturation" behavior would also occur in BFA bodies)? This could also be relevant for explanation of the apparent difference in localization to that described by Steffens et al (2017).
2. (line 146) "3571 long amino acid protein" should be "3571 amino acid long protein"
3. (line 167 ff) The listing of results at the end of the introduction is inappropriate and should be removed.
4. (line 228) YPet is known to form dimers. This does not seem to impair the ability of the SPI-YPet fusion protein to complement the *spi* mutant phenotype, but might be considered in interpreting the localization data.
5. (line 430) "out growths" should be "outgrowths"
6. (line 432) "most of the trichoblasts ... had a high number of root hair bulges" sounds like there were multiple bulges on each trichoblast. I would remove the "a high number of"
7. (line 784 ff) Please stay consistent in calling the microscope "SP8-X"
8. Figure 3: Please define whether these images are single confocal planes or projections of several optical sections.
9. Figure 8: The right-most panel labeled "Tip growth" should also contain the symbol for SPI protein.

Reviewer #3 (Comments for the Author):
The manuscript by Chin et al. describes the identification of a novel spirrig allele. The mutant phenotype is described focusing on root hair positioning and development, localization studies show an apical accumulation through the post-Golgi pathway, and an antagonistic recruitment of SPI and BRK1 to initiating root hairs. The paper is clearly written and the data are generally sound. I have concerns that some conclusions are not fully supported by the data, which are outlined below.
1. The authors conclude that SPI maintains root hair tip focused actin. In my opinion this conclusion is very strong. It is correct that tip-focused actin is strongly reduced in the *spi* mutant and it is correct that SPI has a tip localization in growing root hairs, but I do not see evidence for a direct, causal link between SPI and the actin cytoskeleton during tip growth. One could argue that SPI has a function in tip growth and that disruption of growth has a downstream effect on actin organization.
2. I struggle with the conclusion that SPI and BRK1 are antagonistic. Sure, there is a negative correlation between the presence of the proteins during root hair development, but the failure of BRK1 to disappear from the RHID in *spi* mutants may be caused by general failure to initiate tip growth. Vice versa, if SPI would be antagonistic to BRK1, I would expect an earlier appearance of SPI1 at the RHID in *brk1* , which is not observed. The authors try to get to a better understanding of this observation by analyzing planar polarity and root hair growth defects in different single and double mutants. I wonder how similar the position of the RHID and the ability to initiate a root hair from a RHID is in this study.
3. The actin images are suspiciously beautiful. This suspicion is fueled by the UBQ10 promoter that drives expression of mGFP-Lifeact and circular aggregates of F-actin have been reported previously when using GFP-talin and were shown to be artefacts caused by high expression of the probe. Did the authors make an effort to select moderately expressing lines? And if not, can they show that the probe itself does not cause any defects in actin organization?

Minor points:
4. I applaud the effort to obtain quantitative data of the actin organization and SEC-RFP accumulation (from line 310), nonetheless, the quantification may suffer from the presence of vacuolated sub-apical regions in *spi* root hairs. This issue is evident in Fig. 2E. The oval regions in the inset of figure 2F do not appear to be positioned accurately as they include extracellular space. Have the authors taken these issues into account?
5. I understand that a SEC-RFP gradients are less conspicuous in *spi*, but when secretion is reduced, one would expect accumulation of SEC-RFP in the endomembrane system. Did that authors attempt to quantify this?

TPC2021-RA-00196 Submission received March 10, 2021

Reviewer comments on previously declined manuscript and **author responses:**

GENERAL RESPONSE: We thank the team of editors and the reviewers for this recommendation. We fully agree that the model presented is too speculative and not fully supported by the data. As suggested, we have removed the model from the manuscript. We are also very grateful to the editors for giving us the opportunity to resubmit the paper as a "Breakthrough Report" focusing on the exciting localization data. We agree that the localization of the proteins we report are novel and potentially open new avenues for advancing mechanistic understanding of plant biology. In the revised manuscript, the main Figures (Figure 1-6 – reduced from the original 8 figures) focus on these cell biological and localization studies. In revising the Figures, we have added supporting quantitative analyses of the data requested by some of the reviewers (see below). Also as advised, we have removed all of the genetics data, which we agree are more difficult to interpret without more detailed growth analyses.

Reviewer #1:

Point 1. This manuscript addresses the open question of how actin and endomembrane systems coordinate for tip growth in root hairs, focusing on the SPI protein and the Arp2/3 actin polymerization pathway. The topic is interesting, and the data are, for the most part, well presented. One major area of concern, however, is the integration of data from various mutants into the model presented in Figure 8. The parts of the model that are well supported are:
1. SPI plays a role in tip growth in root hairs.

2. SPI localizes to the root hair tip.
3. SPI localization is temporally anti-correlated with BRK1 localization, and BRK1 remains localized to the growing tip in the absence of SPI.
4. Root hair positioning depends on both SPI and SCAR/WAVE activity, and BRK1 localizes to the RHID.
5. SPI and the Arp2/3 complex both contribute to root hair extension (although it is less clear whether and how SCAR/WAVE plays a role here).

The other conclusions are based on a *brk1* phenotype that is less convincing. In figure 6A, the *brk1* root hairs look just as long as WT, if not longer, and it appears there may be more root hairs in the mutant. Is this image representative? In the quantification (6B), how many plants did the root hairs come from? It seems likely that a few outliers are driving the significance for the difference between WT and *brk1* root hairs. It would be helpful to know if these outliers were found in multiple individuals, or were from the same plant. If *brk1* root hairs are not really different from WT root hairs, the authors might consider altering their conclusion that BRK1 is involved in tip growth: Line 417: "Taken together, our results indicate that SPI is epistatic to BRK1 for root hair planar polarity and tip growth." (To be clear, this concern does not apply to the polarity phenotypes quantified in Fig. 6C which appear robust.)

RESPONSE: Thank you for your comments and enthusiasm for the manuscript. Thank you also for pointing out the datasets that support the model. Despite this, we agree with the reviewer and editors that the model remains too speculative and have followed the advice to remove it altogether.

With regard to the data in the original figure 6 (i.e. *brk1* growth phenotype), we agree that they are more difficult to interpret and less convincing. The editors in their decision letter have advised us to remove the genetic components of the paper and focus primarily on the exciting and novel localization results. Thus, all the data on double mutant studies including those in the original Figure 6 here have been removed from the revised manuscript. Thus, we believe that the issue raised here is now resolved.

Point 2. An alternative hypothesis could be that the SCAR complex is involved in root hair positioning, but not in determining root hair length. This interpretation is consistent with localization data shown in Figure 5A, where SCAR localization is highest during bulge formation and then drops precipitously during root hair growth.

RESPONSE: Thank you for your insight. This comment is also affected by the editor recommendation to remove all of the genetic data from the revised manuscript and for us to focus on the localization data. Because the root hair positioning phenotypes are part of the genetic data, we believe that this comment has been addressed.

Point 3. Finally, the model proposed in Figure 8 includes a lot of details for which the authors have yet to support with data. The authors are up front about the speculative nature of the model, but a simpler model that includes only proteins for which they (or others from the literature) have data seems more appropriate. The authors could also consider showing two models: one showing how SPI might interact in the SCAR pathway, and a second showing their alternative hypothesis that SPI acts in a parallel pathway.

RESPONSE: As noted earlier, the editors have recommended removal of the model presented in the original manuscript submission. We have taken their advice into account and have removed Figure 8 as it is too speculative at the moment.

Point 4. There are a number of places in the paper where the conclusions would be strengthened if the authors were to provide a quantitative analysis of their data:

RESPONSE: We thank the reviewer for the encouragement to provide quantitative analysis of the data to strengthen our conclusions. We feel that the additional quantitative data requested by the reviewer is appropriate given that we have been advised by the editors to focus mostly on the localization data. As detailed below, we provided as much quantitative data requested by the reviewer. Each of the six main Figures and supplemental data now have corresponding quantitative data. Detailed explanations of how these data were acquired are outlined in various sections of the manuscript as well as in our responses below.

Point 5. Fig. 1: What percentage of elongating root hairs show robust SPI-YPet localization at the tip? The localization is very clear: the question is do the authors see this phenotype in all plants, or in just one or two?

Presumably the authors saw the same phenotypes across multiple root hairs and plants, but the data should be provided.

RESPONSE: We are pleased to know that the reviewer agrees that localization of SPI-YPET is very clear and appreciate her/his comment to be more transparent by asking the percentage of elongating root hairs that show robust SPI-YPET localization at the tip. To address this comment, we point out in the figure 1 legend and results text that 100% of elongating root hairs have robust SPI-YPET signal. In making this claim, we indicate in the figure legend the number of seedlings and elongating root hairs examined. To make this even more transparent, we now provide a low magnification movie showing time-lapse sequence of several elongating root hairs expressing SPI-YPET. This movie is provided as a new Supplemental Movie 1 in addition to movies of individual initiating, elongating and maturing root hairs (re-labeled as Supplemental Movies 2 -4, respectively). In the results section of the revised manuscript, we added the following sentences:

"Confocal microscopy of more than 150 root hairs from at least 25 seedlings revealed that SPI-YPet signal was strongest in the tips of rapidly elongating root hairs (Figure 1B and C; Supplemental Movie 1 and 2). Low magnification time lapse movies showed that 100% of rapidly elongating root hairs had robust SPI-YPet signal at the tips (Supplemental Movie 1)."

In the figure legend to panel 1C we added the sentence:

"Images are representative of about 150 root hairs from at least 25 seedlings."

Point 6. Fig. 2: Only one example of a root hair treated with BFA is shown. This is a key result and it seems appropriate to convey how variable this phenotype is. Two additional examples from the elongation zone are shown, but these phenotypes seem qualitatively different than the single root hair example (the puncta are much more obvious in the elongation zone). The authors should quantitate the frequency and intensity of the BFA phenotype in root hairs.

RESPONSE: We thank the reviewer for pointing out that only one example of a root hair treated with BFA is shown. We apologize for this oversight. To address this concern, we have added a low magnification image of control (untreated) and BFA treated root hairs from representative seedlings. This way, the readers are able to see examples of more than one root hair treated with BFA. This low magnification is now labeled as Figure 2A. We kept the high magnification images of single untreated and BFA treated roots hairs (Figure 2B) so that the readers can better see the morphology of the BFA bodies. Because of the new Figure 2A, we have decided remove the data related to BFA bodies in the elongation zone so as to maintain focus on the root hairs.

To address the issue of quantification raised by the reviewer, we counted the number of BFA bodies per root hair from 8 independent plants and added these numbers to the Figure 2A caption. The number of BFA bodies per root hair has been added to the figure legend and results test. Furthermore, as requested by the reviewer, we now provide data on the intensity of the BFA bodies in the root hairs. This was accomplished by measuring the fluorescence intensity of the bright puncta that originated from a BFA body against the fluorescence intensity of the cytoplasm. This new data are presented as a new Figure 2D. In adding the new quantitative data the following sentences were added to the results text:

"The average number of BFA bodies per root hair was 3.69 ± 4.05 (S.D). Quantification of the BFA effect was conducted by obtaining the fluorescence ratio of the SPI-YPet agglomerates to the fluorescence of the root hair cytoplasm that did not contain any agglomerates (Figure 2D, inset). The higher fluorescence ratio of BFA- treated root hairs compared to untreated controls reinforces our qualitative observations of the sensitivity of SPI-YPet to BFA (Figure 2D)."

In the revised Figure 2 legend, we added the following text:

For Figure 2A and B:

"(A) to (B) Low magnification image showing several root hairs expressing SPI-YPet. Note that untreated root hairs maintain tip-focused SPI-YPet while those treated with BFA show an abundance of fluorescence agglomerates (arrows).

For the new quantitative data for panel 2D:

"(D) Box plot of BFA induced agglomerates of SPI-YPet in untreated control root hairs and after treatment with 50

µM BFA. Ratio values were obtained by dividing mean fluorescence in rectangular region in 1 over region 2 (inset). Box limits indicate 25th percentile and 75th percentile, horizontal line is the mean and whiskers display min and max values. Each dot represent individual measurements. Asterisk (***) indicates statistical significance ($p<0.001$) as determined by Student's T-test. Means (n = 8-14 root hairs) ± SE from 8-24 plants. BFA treated plants had an average of 3.7 BFA-induced agglomerates per root hair, with standard deviation of 4.05, whereas control root hairs showed no BFA-induced agglomerates per root hair. "

Point 7. Fig 3Q: Co-localization of SPI and F-actin is an important finding, yet the authors show only a single example, which is not very compelling: actin intensity is seen throughout the cell, so the only way the image could show lack of co-localization would be a depletion of actin where SPI-YFP is. At minimum, additional examples should be provided, but quantitation of the phenotypes would be preferable.

RESPONSE: We are glad that the reviewer considers the co-localization of SPI and tip-focused F-actin as an important finding. The point raised by the reviewer that the only way the image could show a lack of colocalization would be to show a depletion of tip-focused F-actin at a time when SPI-YPet is a very good one. To address this suggestion, we collected a series of images every 10 minutes of elongating root hairs expressing both SPI-Ypet and Lifeact-mRuby for time durations spanning 2 hours. In doing so, we were able to document the decline of tip growth in some sequences. A representative sequence spanning 2 hours is shown in a new Figure 4A. From this figure, one can see that the growing root hair, both SPI-Ypet and Lifeact-m-Ruby have distinct tip-focused gradients (similar to what we have shown in the original Figure 3Q). However, with a series of images, one can now see that upon reaching the 80 - 120 min time point, tip growth has clearly ceased. At these time points, both tip-focused SPI-YPet and Lifeact-mRuby have dissipated. This shows indirectly that maintaining tip focused SPI-YPet is linked tightly to that of the tip-focused F-actin meshwork.

We also thank the reviewer for the suggestion to quantify the data. This was done by obtaining the fluorescence ratio of SPI-YPet and mRuby-Lifeact from at least 3 time lapse sequences. We then analyzed the data by correlation. The new data are presented as a new Figure 4B and C to show how fluorescence measurements were made (Figure 4C) and the new correlation data (Figure 4B). This new data clearly shows that when tip-focused SPI-YPet dissipates from the root hair tip as the root hair ceases growth, the tip-focused F-actin meshwork also dissipates strengthening our conclusion about the relationship between SPI and F-actin in root hair tip growth. The addition of the new Figure 4 and associated quantitative data led to the addition of the following sentences in the results text:

"We next generated plants expressing both SPI-YPet and mRuby-Lifeact so we could correlate SPI and F-actin in growing root hairs. In elongating root hairs of dual labeled seedlings, SPI-YPet and the mRuby-labeled F-actin meshwork overlapped at the tip of actively elongating root hairs (Figure 4A; Supplemental Movie 6). In one time lapse sequence, when the root hair stopped growing at the 80 – 120 min time points, both SPI-YPet and mRuby-labeled F-actin meshwork dissipated from the root tip (Figure 4A). Quantification of both SPI-YPet and mRuby-Lifeact from at least three root hair time lapse sequences revealed that the appearance of both markers at the tip are highly correlated to each other (Figure 4B and C). This provides support that root hair tip-localized SPI is strongly associated with the tip-focused F-actin meshwork, and as such is involved in sustaining normal root hair elongation in coordination with actin. "

The legend for the new Figure 4 is:

"Figure 4. SPI-YPet and mRuby Lifeact co-localizes at the root tip in elongating root hairs.

(A) Time course of a root hair simultaneously expressing SPI-YPet and mRuby-Lifeact. Note that SPI-positive post-Golgi compartments and F-actin meshworks colocalized at the root hair apex (arrows) and dissipated at around the same time (arrowheads) at 80 min. Images are single median optical sections. Bars = 10 µm.

(B) Scatter plot showing correlation analysis of root hair tip mRuby-Lifeact fluorescence and SPI-YPet fluorescence within the same root hair. The mean fluorescence in the rectangle in region 1 divided by the rectangle in region 2 as shown in panel C represents the fluorescence ratio for each reporter. For each ratio value, root hair growth rate was obtained by measuring the displacement of the root hair tip after a 10 min interval. Line shows linear regression fit with R^2 value = 0.725 and $p= 2.127 \times 10^{-8}$. (n = 26 time points from 3 root hair sequences)

(C) Method for obtaining SPI-YPet and mRuby-Lifeact ratios at the root hair tip for data shown in B. A rectangular region of interest at the tip and subapex was used to measure fluorescence."

Point 8. The timecourse data do not indicate how frequently the authors see the patterns shown. Presumably the authors saw the same phenotypes across multiple root hairs and plants, but the data should be provided. At minimum, additional examples should be provided, but quantitation of the phenotypes would be preferable.

RESPONSE: Because of the addition of a new Figure 4 (see above), the original Figure 5 is now a revised Figure 6. We apologize for not providing data on how frequently the patterns of persistent BRK1-YFP

As advised by the reviewer, we now provide quantitative data to better reflect this phenotype is seen (i.e. BRK1-YFP in spi). To do this, we selected root hairs of *brk1* and *spi* expressing BRK1-YFP that were roughly equivalent in length (approximately 30 µm). We then took the ratio of the tip fluorescence to the subapical region of the root hair as shown in panel 6D. The extracted data are shown in new panel 6E. From the data in panel 6E, one can clearly see the persistence of BRK1-YFP at the tip of a slow growing *spi* root hairs that are roughly equivalent size as that of BRK1-YFP in *brk1.* This conclusion is based on the higher tip to apex fluorescence ratio of BRK1-YFP in *spi* when compared to BRK1-YFP in *brk1.* In the figure legend, we indicate that the measurements were made from 3-6 root hairs from 8 different plants. The new quantitative data requested by the reviewer led us to add the following text to the results section:

"To support out visual observations with quantitative data, we selected root hairs of *spi* and *brk1* expressing BRK1-YFP that were of about equal lengths. From these root hairs, the ratio of BRK1-YFP tip fluorescence to subapical fluorescence was obtained (Figure 6D). These analyses showed that BRK1-YFP signal persisted in *spi* as demonstrated by the higher fluorescence ratio (Figure 6E). By contrast, BRK1-YFP in *brk1* root hairs disappeared from RHID when root hairs experienced rapid tip growth and thus did not exhibit fluorescence gradient at the root tips (Figures 6D and E). "

The new panels in Figure 6 led to the addition of the following sentences in Figure 6 legend:

"(D) Method for quantification of BRK1-YFP signal persistence in the root tip of spi. Root hairs of *brk1* and *spi* expressing BRK1-YFP that were about 30 mm in length were selected. Rectangular region of interests (1 and 2) were drawn to obtain fluorescence values. Ratio values were obtained by dividing mean fluorescence in rectangle region in 1 over region 2 used to plot data in panel E.

(E) Box plot of BRK1-YFP root hair tip gradient expressed as fluorescence ratio. Box limits indicate 25th percentile and 75th percentile, horizontal line is the mean and whiskers display min and max values. Asterisk (**) indicates statistical significance ($p<0.01$) as determined by Student's T-test. Means (n = 3-6 root hairs) ± SE from 8 plants. Only root hairs of similar length were compared. The average root hair length for *brk1* was 37.28 µm (standard deviation 18.05 µm) and for spi5 was 38.74 µm (standard deviation 18.34 µm)."

Point 9. There is something unusual going on with the error bars (standard error) for some of the samples shown Figures 6B and 7C. These graphs are plotted on a log scale, but the error of some of the samples are the same height above and below them mean. On a log scale, we would expect the error to be shorter above the mean and longer below to reflect the same number.

RESPONSE: Because data referred to by the reviewer are related to the genetics, Figures 6B and 7C from the original submission have been removed as suggested by the editors. Thus, we believe this is no longer an issue.

Point 10. Fig. 7 is central to the take home message of the paper, and the authors should include all the data points as a bee swarm plot or similar, particularly for panel B as the difference between WT and *brk1* is central to their model.

RESPONSE: As previously mentioned, we have removed Figure 7 following the suggestion of the editors.

<u>Reviewer #2:</u>

Point 1. line 273 and 304 ff) The authors mention slow-growing root hairs in *spi* mutants. It would be helpful to characterize these growth rates so they could be compared to, for example, the rates shown in Figure 1D. This analysis could also be extended to an explicit comparison on growth rate and actin fluorescence. The authors already have data on the latter (Figure 3P).

RESPONSE: Thank you for your insight and suggestions. We agree that presenting data as growth rates would be more informative. Thus, we have replaced root hair length data in the original supplemental Figure 2C with root hair

growth rates.

A more important point raised is to provide a more explicit comparison between growth rate and actin. We believe that this issue has now been addressed based on our response to a comment by reviewer 1 and the addition of a new Figure 4. As noted above and in the new Figure 4, we show that tip-focused F-actin is not maintained when root hair tip growth occurs, but also when tip-focused SPI-YPet is present (new Figure 4B). The new Figure 4B legend states:

(B) Scatter plot showing correlation analysis of root hair tip mRuby-Lifeact fluorescence and SPI-YPet fluorescence within the same root hair. The mean fluorescence in the rectangle in region 1 divided by the rectangle in region 2 as shown in panel C represents the fluorescence ratio for each reporter. For each ratio value, root hair growth rate was obtained by measuring the displacement of the root hair tip after a 10 min interval. Line shows linear regression fit with R^2 value = 0.725 and p= 2.127 x 10^{-8}. (n = 26 time points from 3 root hair sequences)

Note that the underlined section states that root hair growth rate was obtained to correspond to each fluorescence ratio measurement for SPI-YPet and Lifeact-mRuby.

Point 2. (line 390) The observation of prolonged persistence of BRK1-YFP in elongating root hairs of the *spi* mutant is very interesting. I am wondering whether the *spi* root hairs can only grow when BRK1 is maintained at the root hair tip. A prediction from this hypothesis would be that all actively growing *spi* root hairs have BRK1-YFP signals at their tip, and that there may be a positive correlation of this signal with growth rate (unlike in WT). This test could indicate whether ARP2/3 activation could compensate for the loss of SPI. I assume the authors already have the necessary to data to perform such an analysis.

RESPONSE: Thank you for raising these points. They are all very good and certainly are possible scenarios. We believe, however, from observing many root hairs expressing BRK1-YFP in *spi* is that even non-growing, aborted *spi* root hairs maintain a persistent BRK1-YFP signal (see Figure 6B). Also, not all actively, slow growing *spi* root hairs have persistent BRK1-YFP at their tips. These qualitative observations prompted reviewer one to provide quantitative data to better demonstrate the variability of BRK1-YFP persistence in spi. The new quantitative data are shown in a new Figure 6D and E. Based on this analysis, it is more likely that slow growing or non-growing *spi* root hairs display more of a persistent BRK1-YFP signal.

Point 3. (line 275) The authors present data in Figure 2D that suggest that a defect in accumulation of exocytic vesicles at the root hair tip of *spi* mutants. This is difficult to reconcile with the ongoing (albeit slow) growth of these root hairs. It would be helpful to test with another marker such as FM4-64 whether the accumulation of vesicles at the tip of growing *spi* root hairs is really gone.

RESPONSE: Thank you for bringing this to our attention. As requested, we have conducted additional FM 1-43 dye experiments in wild-type and *spi* root hairs. The data is presented as a new Supplemental Figure 4). Results from FM 1-43 dye corroborated our SEC-RFP results. Consistent with SEC-RFP assays, spi-5 root hairs exhibited reduced vesicle accumulation at the tip as shown by reduced FM incorporated membrane fluorescence. As a result of the new data, we added the following sentences to the results text:

"These results were corroborated with FM 1-43 dye uptake results, in which *spi* root hairs showed significantly reduced tip focused FM 1-43 gradient (Supplemental Figure 4A to C). Altogether, the loss of tip-focused secretion indicated defects in tip-directed bulk flow exocytosis in *spi* mutants."

The new Figure legend for Supplemental Figure 4 reads:

"Supplemental Figure 4. FM-1-43 uptake assays in wild-type and *spi* root hairs.

(A) and (B) Distinct FM-1-43 fluorescence accumulates at the tips of wild type (arrows), but not *spi* root hairs

(C) Quantification of FM1-43 gradients in wild type and *spi* root hair tips. Fluorescence ratio was obtained by marking oval regions of interests as shown in panel B and dividing 2 over 1. Box limits indicate 25th percentile and 75th percentile, horizontal line is the mean and whiskers display min and max values. Asterisk (*) indicates statistical significance (p=0.02) as determined by Student's T-test. n=16 root hairs from at least 10 independent seedlings."

Point 4. (line 457) I don't think the statements that *arp2*, *arp3*, and *brk1* "leaned more toward wild-type sensitivity" and that the *spi brk1* double mutant "was more similar to spi" are justified. The single mutants have growth reductions that

are about half-way between WT and *spi*, and the *spi brk1* growth inhibition seems to be additive of the single mutants. Based on the data in Supplemental Figure 6B, it seems that *spi* is epistatic to *arp2* and *arp3* for LatB inhibition of primary root growth, but that *spi* and *brk1* function additively. These are different genetic interactions from those found for the root hair phenotypes, but this does not invalidate the conclusions of the authors for root hair growth.

RESPONSE: Thank you for letting us know. We agree that these are good and valid points. However, because the data referred to are related to the genetics work, these data have been removed as suggested to us by the editors to make the paper more suitable for a "breakthrough report" and avoid the uncertainty from the genetics work.

Reviewer #3:

Point 1. The authors conclude that SPI maintains root hair tip focused actin. In my opinion this conclusion is very strong. It is correct that tip-focused actin is strongly reduced in the *spi* mutant and it is correct that SPI has a tip localization in growing root hairs, but I do not see evidence for a direct, causal link between SPI and the actin cytoskeleton during tip growth. One could argue that SPI has a function in tip growth and that disruption of growth has a downstream effect on actin organization.

RESPONSE: Thank you raising this point. We agree that direct causal links between SPI and F-actin have yet to be established. We agree that the disruption of tip growth due the absence of SPI could have a downstream effect on actin organization. We have added this point in paragraph 2 of the discussion. The added sentences read:

"Although the observation that the presence of tip-focused SPI-YPet was strongly correlated with maintenance of the tip-focused F-actin meshwork, direct causal links between SPI and F-actin have yet to be established. An alternative explanation is that tip growth induced reduction due to the absence of SPI could lead to downstream, indirect effects on actin organization."

Point 2. I struggle with the conclusion that SPI and BRK1 are antagonistic. Sure, there is a negative correlation between the presence of the proteins during root hair development, but the failure of BRK1 to disappear from the RHID in *spi* mutants may be caused by general failure to initiate tip growth. Vice versa, if SPI would be antagonistic to BRK1, I would expect an earlier appearance of SPI1 at the RHID in *brk1* , which is not observed. The authors try to get to a better understanding of this observation by analyzing planar polarity and root hair growth defects in different single and double mutants. I wonder how similar the position of the RHID and the ability to initiate a root hair from a RHID is in this study.

RESPONSE: Thank you for bringing up this point. Although there is indeed a negative correlation between the presence of BRK1-YFP and rapid tip-growth, we agree that the failure of BRK1 to disappear from the RHID in *spi* mutants may be caused by general failure to initiate tip growth. As suggested, we have removed the word antagonistic from the revised manuscript. The point raised about the general failure to initiate tip growth in the *spi* mutants as a cause for the persistent BRK1-YFP signal has also been added to the second to the last paragraph of the discussion. The added sentences are posted below:

"Alternatively, the failure of BRK1 to disappear from the RHID in *spi* mutants may be caused by general failure to initiate tip growth. Future studies will require subjecting root hairs to conditions that prematurely terminate tip growth to determine if BRK1 signals persist."

Point 3. The actin images are suspiciously beautiful. This suspicion is fueled by the UBQ10 promoter that drives expression of mGFP-Lifeact and circular aggregates of F-actin have been reported previously when using GFP-talin and were shown to be artefacts caused by high expression of the probe. Did the authors make an effort to select moderately expressing lines? And if not, can they show that the probe itself does not cause any defects in actin organization?

RESPONSE: We appreciate the comment about actin images being beautiful. The quality of images is because they were obtained with a spinning disc confocal microscope using a 100x oil immersion objective. The quality of spinning disc images is much higher than those obtained with a point scanning confocal microscope. The point about high expression of F-actin probes causing artefacts is indeed a valid point. Yes the lines we used are moderately expressing. In fact it has been shown that driving expression of F-actin reporters with the UBQ10 promoter is less intrusive than the 35S promoter. To address this concern further, we have modified Figure 3 by

adding a new panel Figure 3Q. n this new panel, we show that expression of UBQ10:mGFP-Lifeact construct did not affect root hair growth rate in wild type and *spi* lines. As shown in Figure 3Q root hair growth rates were the same wild type and *spi* with and without expressing the UBQ10:mGFP-Lifeact. As a result of the new Figure 3Q, the following has been added to the Figure 3 legend:

"(Q) Comparison of root hair growth rates between wild type and *spi* lines with their corresponding live F-actin reporter lines,UBQ10: mGFP-Lifeact,. Box limits indicate 25th percentile and 75th percentile, horizontal line is the mean and whiskers display min and max values. Letters indicates statistical significance ($p < 0.05$) as determined by one-way ANOVA. Means (n = 4-5 root hairs) ± S.E from 1-2 plants."

The text below was also added to the results section to reflect the new Figure 3 panel"

"To ensure that the mGFP-Lifectact probe did not interfere with normal root hair elongation or F-actin organization, we compared the growth rates of wild type and *spi* with and without the reporter. The root hair growth rates of wild-type root hairs without the mGFP-Lifeact probe was not significantly different from wild-type root hairs expressing the reporter. Similarly, the growth rate of *spi* expressing mGFP-Lifectact was not was not significantly different from *spi* without the reporter (Figure 3Q)."

TPC2021-RA-00196 1st Editorial decision – *accept with minor revision* March 15, 2021

On the basis of the advice received, the board of reviewing editors would like to accept your manuscript for publication in The Plant Cell. This acceptance is contingent on revision based on the comments of our editors. In particular, please consider the following:

Comment 1. line 104 - please reword as "best" is a very strong word. I might suggest the following wording: "one of the most well characterized"

RESPONSE: We have replaced the word to "one of the most well characterized" as recommended.

Comment 2. line 128 - *Physcomitrella patens* is now officially *Physcomitrium patens*. I would suggest using *Physcomitrium* (formerly *Physcomitrella*) *patens* the first time and then simply using *P. patens* in the rest of the document.

RESPONSE: We have replaced the word *Physcomitrella* to *Physcomitrium* and referred the plant as *P. patens* onwards.

Comment 3. lines 252-257 describe the effect of BFA treatment. While this description is true for Arabidopsis roots, we refer you to the following reference, "https://www-sciencedirect-com.dartmouth.idm.oclc.org/science/article/pii/S1360138508001799", which carefully describes how there are tissue specific effects of BFA. Please modify the text to reflect how the case in roots is somewhat special.

RESPONSE: We have expanded our description of BFA treatment and included the additional sentences: In Arabidopsis roots, BFA binds to the ARF-GEF, GNOM that is localized to early endosomes in the endocytic pathway, and possibly also in the trans-Golgi network (TGN)(Robinson et al., 2008). Consequently, BFA treatment in Arabidopsis roots causes the formation of TGN/ endosomal agglomerations called BFA-induced compartments.

Comment 4. lines 264-266 - It is a little unclear why this localization is considered to be post-Golgi vesicles. Perhaps the localization in addition to accumulation in BFA-induced compartments point to post-Golgi compartments.

RESPONSE: We agree that BFA- induced compartments can also be endosomal and have reworded post-Golgi vesicles as post-Golgi compartments.

Comment 5. line 259 - perhaps it would be best to not call these compartments BFA bodies (BFA-induced compartments works) as they have not been labeled with FM4-64, for example to demonstrate that they are in fact the same compartments.

RESPONSE: We have renamed BFA-induced bodies as BFA-induced compartments.

Comment 6. line 274 - should "tip-directed exocytosis" actually be "tip-directed endocytosis"?

RESPONSE: We have removed the "tip-directed exocytosis" and replaced it with "endocytosis".

Comment 7. line 505 - please change "seedling plants" to seed plants.

RESPONSE: We have changed the wording to seed plants.

Comment 8. For the ANOVA analysis in figure 3, please specify if a post-hoc test was used.

RESPONSE: In the figure legend for Figure 3, we have specified that Tukey's post-hoc test was used.

Comment 9. Please describe statistical methods (T-tests and ANOVA seem to be missing) used fully in the materials and methods.

RESPONSE: We have included a description of how we performed t-tests and ANOVA under Materials and Methods section : "Pairwise t-tests and ANOVA tests were performed using lsmeans package (Lenth, 2016) in R (R Core Team, 2019) ."

**TPC2021-RA-00196R1 1st Revision received**	**March 31, 2021**

**TPC2021-RA-00196R1 2nd Editorial decision – *acceptance pending***	**March 31, 2021**

We are pleased to inform you that your paper entitled "Spatial and Temporal Localization of SPIRRIG and WAVE/SCAR Reveal Roles for These Proteins in Actin-Mediated Root Hair Development" has been accepted for publication in The Plant Cell, pending a final minor editorial review by journal staff. At this stage, your manuscript will be evaluated by a Science Editor with respect to its presentation of scientific content, compliance with journal policies, and presentation for a broad readership.

**Final acceptance from Science Editor**	**April 13, 2021**
