# Supplementary figures and images for: Spatial and temporal localization of SPIRRIG and WAVE/SCAR reveal roles for these proteins in actin-mediated root hair development
Source: Plant Cell. 2021 Apr 20;33(7):2131–48. doi: 10.1093/plcell/koab115 (PMC8364238; doi:10.1093/plcell/koab115)

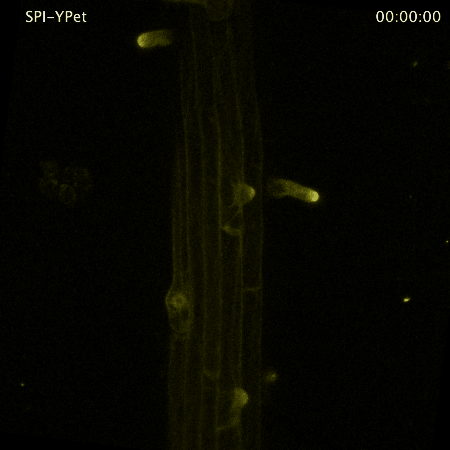

Supplement: koab115_Supplementary_Data [file koab115_supplementary_data.zip › tpc.00196.2021-s02.gif]

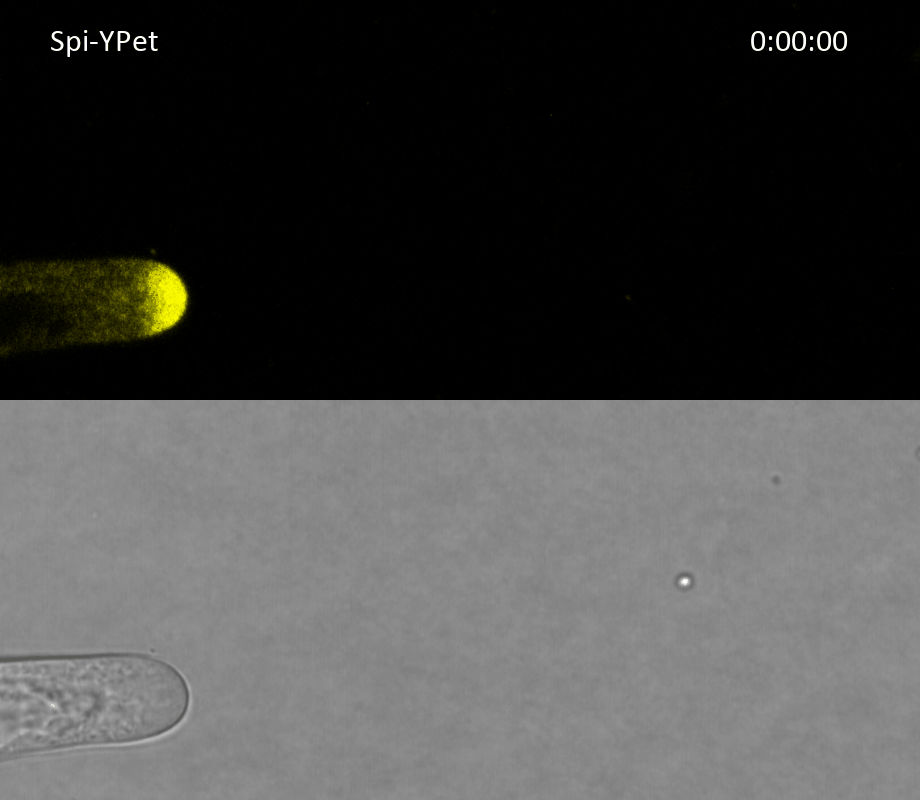

Supplement: koab115_Supplementary_Data [file koab115_supplementary_data.zip › tpc.00196.2021-s03.gif]

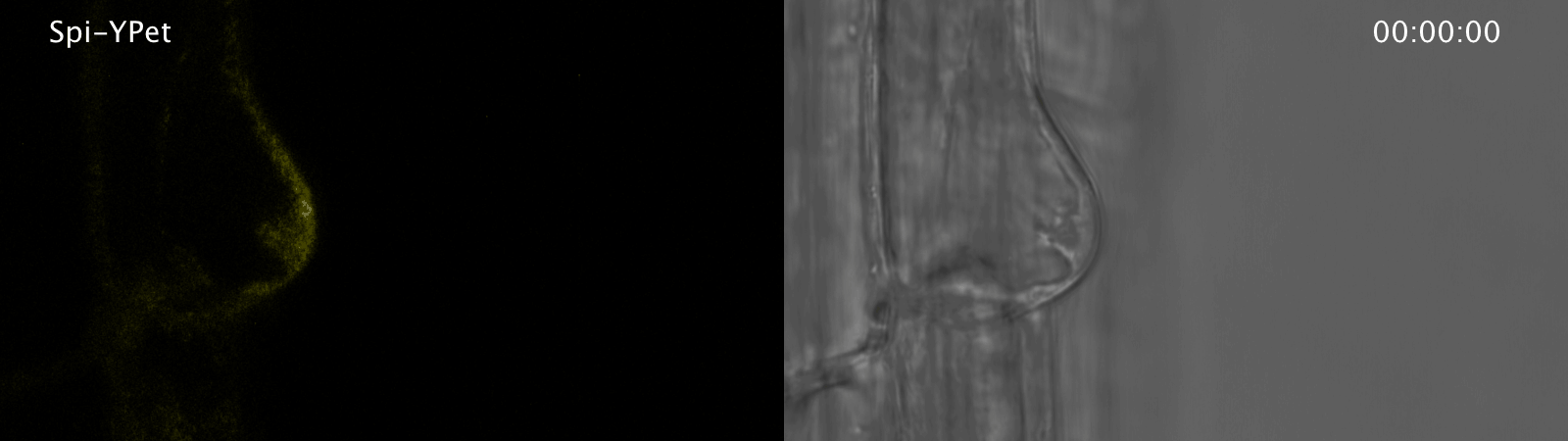

Supplement: koab115_Supplementary_Data [file koab115_supplementary_data.zip › tpc.00196.2021-s04.gif]

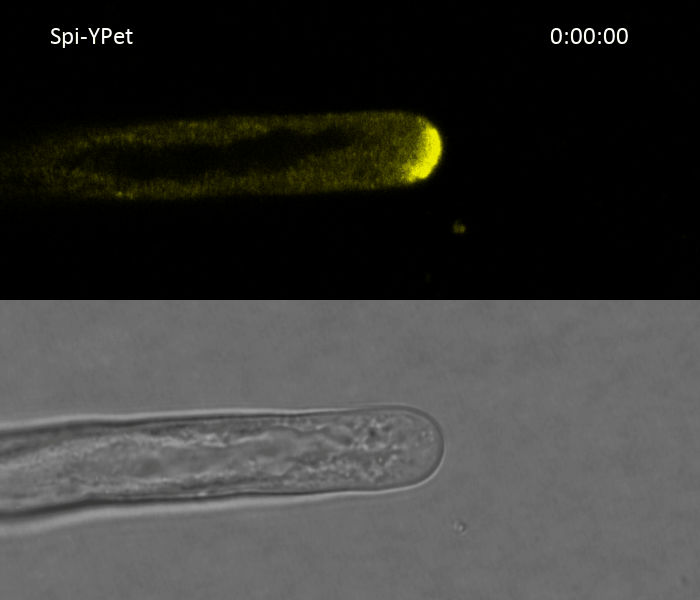

Supplement: koab115_Supplementary_Data [file koab115_supplementary_data.zip › tpc.00196.2021-s05.gif]

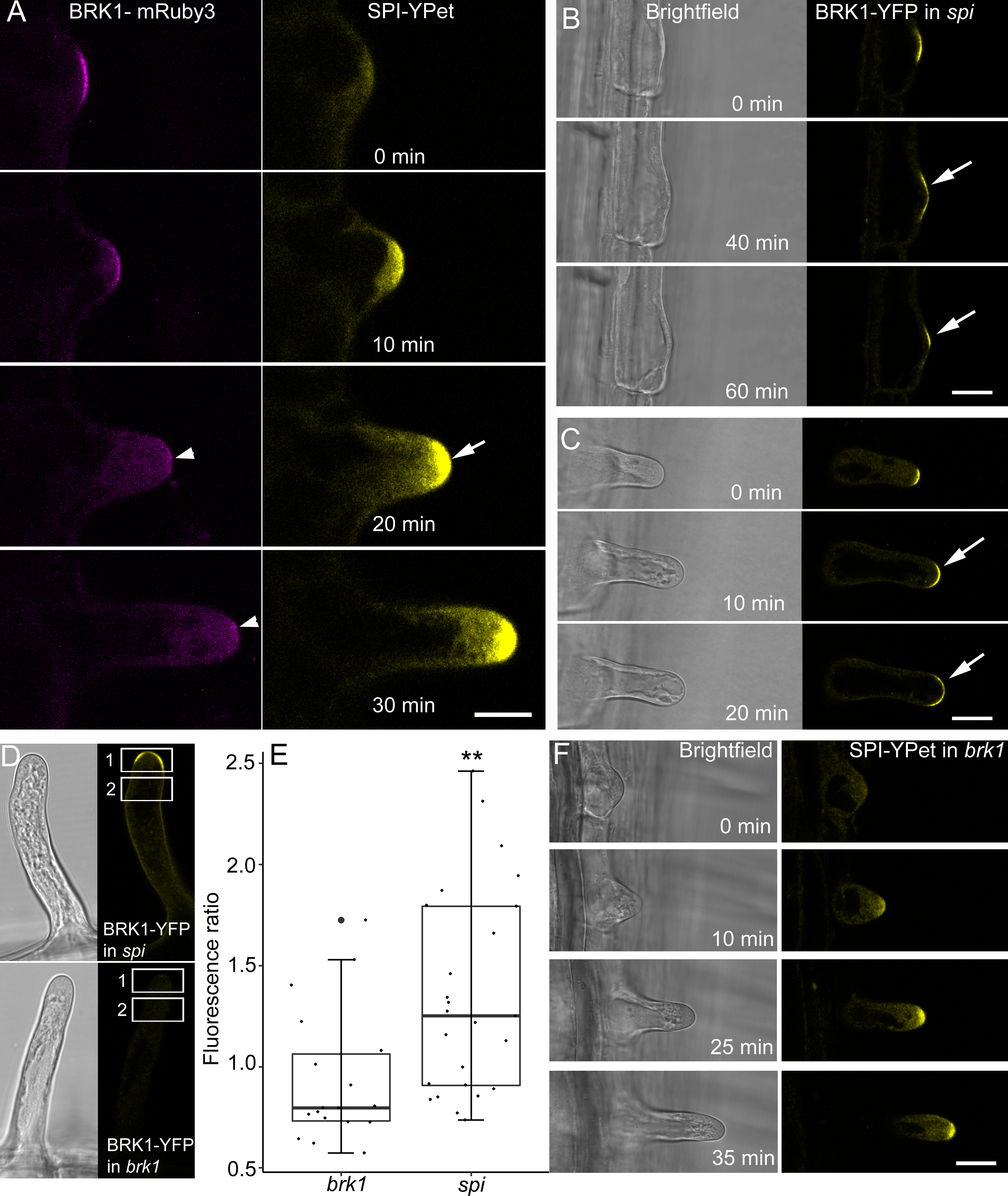

Supplement: koab115_Supplementary_Data [file koab115_supplementary_data.zip › tpc.00196.2021-s07.tif]

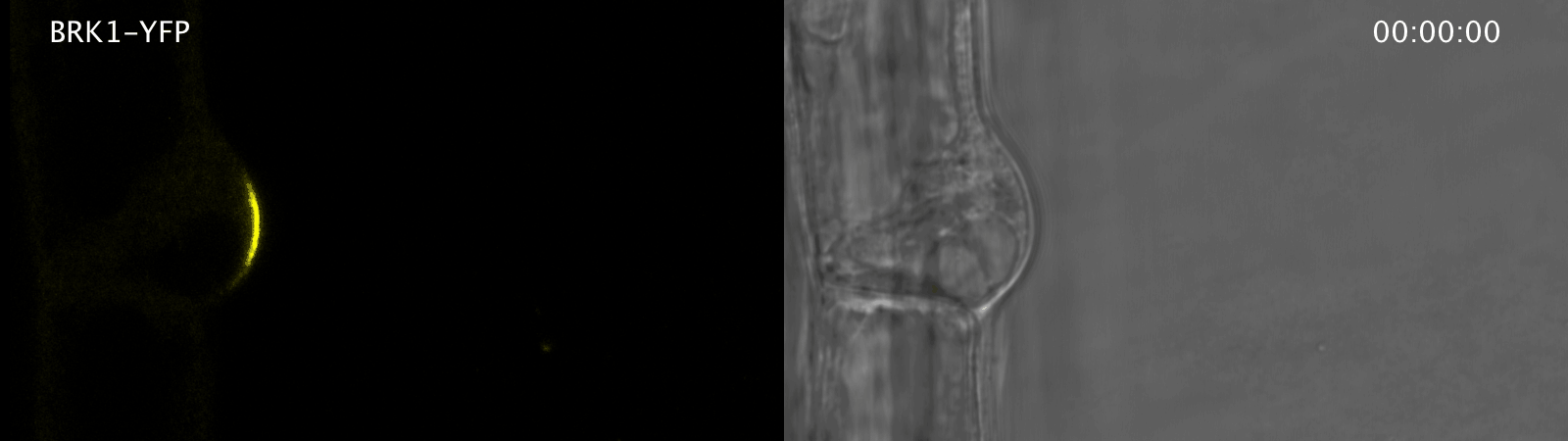

Supplement: koab115_Supplementary_Data [file koab115_supplementary_data.zip › tpc.00196.2021-s08.gif]

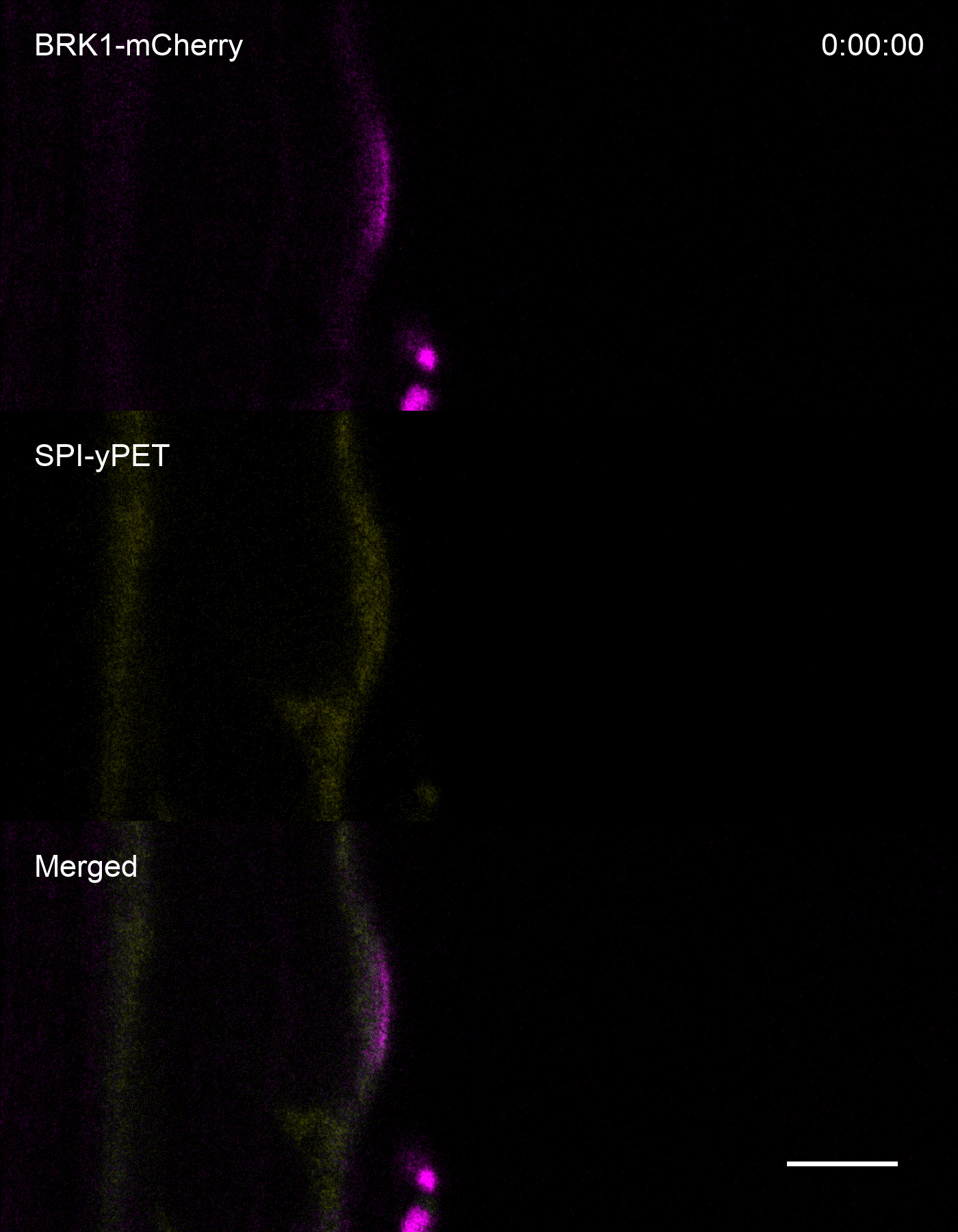

Supplement: koab115_Supplementary_Data [file koab115_supplementary_data.zip › tpc.00196.2021-s09.gif]

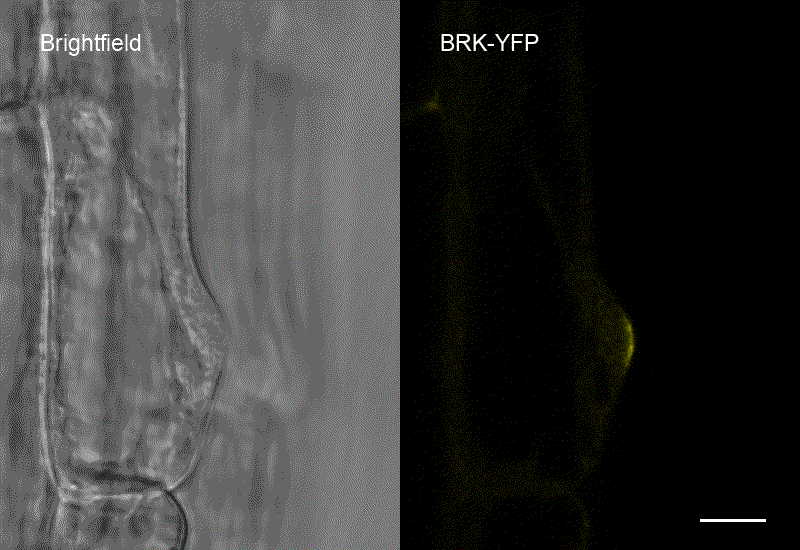

Supplement: koab115_Supplementary_Data [file koab115_supplementary_data.zip › tpc.00196.2021-s10.gif]
